# A randomized controlled trial on preweaning morbidity, growth and mortality in Holstein heifers fed a lacteal-derived colostrum replacer or pooled maternal colostrum

**DOI:** 10.1186/1746-6148-9-168

**Published:** 2013-08-21

**Authors:** Sharif S Aly, Patrick Pithua, John D Champagne, Deborah M Haines

**Affiliations:** 1 Veterinary Medicine Teaching and Research Center, School of Veterinary Medicine, University of California, Davis, 18830 Road 112, Tulare, CA 93274, USA; 2 Department of Population Health and Reproduction, School of Veterinary Medicine, University of California, One Shields Avenue, Davis, CA 95616, USA; 3 Department of Veterinary Medicine & Surgery, Veterinary Medicine Teaching Hospital, College of Veterinary Medicine, University of Missouri, 900 E. Campus Drive, Columbia MO 65211, USA; 4 Department of Veterinary Microbiology, Western College of Veterinary Medicine, University of Saskatchewan, Saskatoon SK S7N 5A2, Canada; 5 The Saskatoon Colostrum Co. Ltd, 30 Molaro Place, Saskatoon SK S7K 6A2, Canada

**Keywords:** Lacteal-derived colostrum replacer, Pooled maternal colostrum, Calf morbidity, Daily weight gain

## Abstract

**Background:**

The objective of this randomized controlled trial was to determine the effect of feeding a commercial lacteal-derived colostrum replacer (CR) or pooled maternal colostrum (MC) on preweaning morbidity, growth and mortality in Holstein heifer calves. A total of 568 calves were randomly assigned to be fed either 3.8 L of pooled MC or two doses (200 g IgG) of a CR. Calves were monitored daily for preweaning morbidity until weaning at 60 d old. Birth and weaning weights were measured to estimate growth rates.

**Results:**

Calves fed CR were significantly less likely to be affected with a diarrhea event (OR = 0.58; 95% CI, 0.38 to 0.88; P value = 0.011) and had a higher rate of daily weight gain (0.051 kg/day; 95% CI, 0.03 to 0.08; P value <0.001) compared to calves fed pooled MC. Use of lacteal-derived colostrum replacer was not significantly associated with respiratory disease (OR = 1.01; 95% CI 0.67 to 1.51; P value = 0.974 ), omphalitis (OR = 0.93; 95% CI 0.06 to 14.86; P value = 0.956), or mortality (HR = 0.71; 95% CI 0.27 to 1.92; P value = 0.505) in the study calves.

**Conclusions:**

The lacteal-derived CR fed at the study dose was a viable colostrum alternative in the event of poor quality pooled MC for the prevention of preweaning diarrhea and resulted in higher growth rates in comparison to calves fed pooled MC in the study herd.

## Background

Bovine maternal colostrum (MC) contains immune factors that provide a critical first line of defense against a variety of infectious pathogens to which calves become exposed after birth [1]. There is consensus in the literature that at least 3.8 L of good-quality MC (IgG >50 g/L and Total Plate Counts, TPC <100,000 cfu/mL) should be administered to prevent failure of passive transfer (FPT) of immunity, a condition characterized by serum IgG < 10 g/L ≥ 24 h post colostrum ingestion in calves [2-4]. Failure of passive transfer of immunity is a well-recognized risk factor for increased preweaning calf morbidity and mortality [5-8]. The condition has also been associated with milk yield losses and decreased longevity in lactating adult cows [5,6].

Adequate intake of MC is critical for preventing FPT and its negative consequences on calf health and future production performance. Several studies have confirmed the presence of bovine specific pathogens (e.g. *Escherichia coli*, *Salmonella* sp., *Mycoplasma* sp., bovine leukemia virus, and *Mycobacterium avium* subsp. *paratuberculosis*) in MC suggesting that feeding raw MC is an early means by which newborn calves can become exposed to such pathogens [9-15]. In addition, negative correlations have been reported between bacterial contamination levels in colostrum and post-feeding serum IgG concentrations [3,16]. Hence, feeding colostrum of inferior quality (IgG < 50 g/L and TPC > 100,000 cfu/mL) can exacerbate the risk of FPT in calves and compromise preweaning performance [3].

Colostrum replacers derived from either spray-dried bovine plasma or spray-dried cow colostrum are formulated to provide ≥ 100 g of IgG to calves when ingested at the replacer dose [17]. Specific heat treatment must be utilized along with spray-dry processing of the plasma or colostrum to effectively destroy all pathogens present in MC [18]. Therefore, CR products have the potential to limit calf exposure to bovine pathogens that may be shed or contaminate MC and are indicated for calves when quality or quantity (or both attributes) of raw MC may be compromised.

Previous IgG absorption studies on various CR products yielded conflicting results [19-22]. In addition, studies that report a positive association between bovine lacteal or serum based CR products and adequate transfer of immunity in calves rarely include an assessment of preweaning morbidity and performance [19-21]. In one Minnesota study, 93% of calves fed a plasma-derived CR product (125 g of IgG) after birth experienced FPT compared with 28% of calves fed MC [19]. Even so, preweaning morbidity and mortality risks did not differ between groups in this study [22]. Several studies reported that feeding a higher IgG mass (≥ 200 g of IgG) delivered using CR products resulted in greater IgG absorption and lower FPT risks [19,20,23]. However, these studies did not evaluate post-administration performance in terms of preweaning morbidity and mortality risks, feed intake, and weight gain for calves fed larger masses of IgG in the CR products evaluated.

We recently completed a study comparing IgG and serum total protein (TP) in calves fed pooled MC or lacteal-derived CR [24]. In that study, serum TP and IgG concentrations at approximately 24 hours post-colostral intake were significantly lower for calves fed pooled MC (TP = 4.77 g/dL, SD = 0.55; IgG = 7.50 g/L, SD = 5.0) compared to calves fed CR (TP = 5.50 g/dL, SD = 0.52; IgG = 15.15 g/L, SD = 4.75). Colostrum IgG concentration and mass fed were significantly higher in the CR (71.4 g/L; 200 g, respectively) compared with MC group (21.08 g/L, SD = 9.77; 79.79 g, SD = 36.96, respectively). The apparent efficiency of IgG absorption (AEA) was similar for calves fed MC (28.79%, SD = 2.098) or CR (26.98%, SD = 1.65). A significantly higher proportion of calves fed MC (188/269 [70%]) suffered from FPT (serum IgG < 10 g/L) compared with calves fed CR (32/292[11%]) (*P* < 0.001). However, producers are often interested not only in passive transfer of immunity in calves, but also in preweaning health and growth rates. The objective of this randomized trial was to determine the effect of feeding a higher dose of a commercial lacteal-derived CR or pooled MC on preweaning morbidity, daily weight gain and mortality in Holstein heifer calves.

## Results

### Descriptive data

In total, 568 newborn calves were enrolled in the study. Of these, 273 (48%) were fed pooled MC while 295 (52%) were fed lacteal derived-CR. Calves did not significantly differ in birth weight, precolostral serum IgG or total protein between trial groups (*P* value > 0.05). In addition, calves fed MC or CR did not differ in time to separation from dam (2.35 hours (SD = 2.42) and 2.34 (SD = 2.42), respectively) and time to feeding colostrum (3.99 hours (SD = 2.31) and 4.12 hours (SD = 2.25), respectively).

Preweaning health and mortality events and the mean birth and weaning weights are summarized in Table 1. The proportion of calves with diarrhea, and those treated with antibiotics were significantly higher for calves fed pooled MC compared with calves fed lacteal-derived CR. However, the proportions of calves with respiratory disease, omphalitis or those that died in the preweaning period were similar between groups (CR vs. pooled MC, Table 1). The preweaning mean daily weight gain was significantly higher (P < 0.0001, Table 1) in calves fed lacteal-derived CR compared with calves fed pooled MC.

**Table 1:** Comparison of preweaning morbidity, growth and mortality in a randomized trial of the effect of feeding a lacteal-derived colostrum replacer (CR) or pooled maternal colostrum (MC) in calves

	**Treatment group**								**P value**
	**Lacteal-derived CR**				**Pooled MC**				
	**Estimate**	**n**	**95% Confidence interval**		**Estimate**	**n**	**95% Confidence interval**		
			**Lower**	**Upper**			**Lower**	**Upper**	
Number of calves enrolled		295				273			
Preweaning health events									
Diarrhea (%)	15.9	47	11.8	20.1	24.5	67	19.4	29.6	0.011
Respiratory disease (%)	21.4	63	16.7	26.0	21.2	58	16.4	26.1	0.974
Omphalitis (%)	0.3	1	0.0	1.0	0.4	1	0.0	1.1	0.956
Treatment with antibiotics (%)	33.6	99	28.2	38.9	43.2	118	37.3	49.1	0.018
No health events or treatments (%)	63.4	187	57.9	68.9	54.2	148	48.3	60.1	0.026
Preweaning mortality/100 calves, 60-day follow-up period	2.4	7	0.6	4.1	3.3	9	1.2	5.4	0.506
Mean birth weight (kg)	39.1	293	38.5	39.7	39.4	269	38.8	40.1	0.452
Weaning weight (kg)	58.9	275	57.9	59.8	57.0	248	56.0	57.9	0.006
Mean daily weight gain (kg/day)	0.405	274	0.388	0.422	0.355	247	0.337	0.372	<0.001

### Multivariable models

Heifer calves fed lacteal-derived CR were significantly less likely to experience a preweaning event of diarrhea (*OR* = 0.58; 95% CI = 0.38 to 0.88) (Table 2). Similarly, calves fed lacteal-derived CR were less likely to be treated with antibiotics during the preweaning period (*OR* =0.66; 95% CI = 0.47 to 0.93), compared with calves fed pooled MC. While calves fed 200 g IgG in lacteal-derived CR (vs. pooled MC) at birth experienced significantly higher preweaning mean daily weight gain by 0.051 kg/d (95% CI = 0.026 to 0.075; *P* value < 0.001), the risk of respiratory disease and omphalitis was not dependent on the type of colostrum fed (Table 2). In addition, the Hazard Rate Ratio (HRR) estimated from the Cox proportional hazard model showed that there was no significant effect of type of colostrum fed on the hazard for mortality (HRR = 0.71; 95% CI = 0.27 to 1.92; P = 0.505).

**Table 2:** Final model estimates for preweaning morbidity and mortality events in a randomized trial of the effect of feeding a lacteal-derived colostrum replacer (CR) compared to pooled maternal colostrum (MC) in calves

**Outcome**	**Type of colostrum**	**Number of calves**		**β coefficient (SE)**	**P value**	OR* (95% CI)
		**At risk**	**Events**			
Diarrhea	Lacteal-derived CR	295	47	-0.540 (0.212)	0.011	0.58 (0.38 to 0.88)
	Pooled MC	273	67	Reference	–	
Respiratory disease	Lacteal-derived CR	295	63	0.007 (0.205)	0.974	1.01 (0.67 to 1.51)
	Pooled MC	273	58	Reference	–	
Omphalitis	Lacteal-derived CR	295	1	-0.078 (1.417)	0.956	0.93 (0.06 to 14.86)
	Pooled MC	273	1	Reference	–	
Treatment with antibiotics	Lacteal-derived CR	295	99	-0.410 (0.174)	0.018	0.66 (0.47 to 0.93)
	Pooled MC	273	118	Reference	–	
Healthy calves**	Lacteal-derived CR	295	187	0.380 (0.171)	0.027	1.46 (1.05 to 2.05)
	Pooled MC	273	148	Reference	–	

* OR is the ratio of the odds of outcome in calves fed lacteal-derived CR to the odds of outcome in calves fed pooled maternal colostrum.

** Calves raised from birth to weaning without morbidity or treatment with antibiotics.

Based on findings from this study, we estimate that routine feeding of 200 g of IgG (formulated as the current lacteal-derived CR powder) instead of pooled-MC could eliminate approximately one-fifth of the cases of diarrhea (Population Attributable Fraction, PAF = 0.21; 95% CI = 0.03 to 0.35) and approximately one-tenth of the number of antibiotic-treated calves (PAF = 0.12; 95% CI 0.02 to 0.22) in this population (Table 3).

**Table 3:** Population Attributable Fraction (PAF) for feeding raw pooled maternal colostrum compared to a lacteal-derived colostrum replacer at birth to Holstein heifer calves on a California dairy on preweaning diarrhea and treatment with antibiotics in a randomized trial

	**PAF**		
**Item**	**Estimate (%)**	**95% Confidence interval**	**P value**
Diarrhea	21	3.3 to 35	0.021
Treated with antibiotics	12	1.5 to 22	0.026

## Discussion

As reported elsewhere [24], a significantly higher proportion of calves fed pooled-MC (70%) suffered from FPT (serum IgG < 10 g/L) in comparison to calves fed lacteal-derived CR (11%). Failure of passive transfer of immunity is a well-recognized predictor for increased preweaning calf morbidity [5-8]. It is therefore not surprising that calves fed raw pooled MC in this study were more likely to be treated with antibiotics or experience preweaning event of diarrhea compared with calves fed lacteal-derived CR in the study herd.

Bovine specific pathogens (*Salmonella* sp., *Escherichia coli*, *Mycoplasma* sp.) are frequently shed in colostrum of infected fresh cows either during an epidemic or intermittently during the peripartum period [3]. It is possible that the higher proportion of diarrhea in preweaned calves fed pooled MC (vs. lacteal-derived CR) was a consequence of direct ingestion of such pathogens in pooled MC. However, this theory could not be further investigated given that culture of the pooled MC samples for specific pathogens (example *Salmonella* sp., *Escherichia coli*, *Mycoplasma* sp.) was not performed in the current study.

Our results indicate that only 21% of diarrhea cases and 12% of antibiotic-treated calves would be eliminated in this population if the pooled MC feeding program was replaced by 200 g IgG/ calf delivered in a lacteal-derived CR product. This should not be surprising given that in replacement heifer rearing, improved preweaning health is a function of several factors including reducing calf exposure to risk factors (infectious agents inclusive). This can be accomplished through proper housing, ventilation, and general hygiene; increasing levels of specific and non-specific immunity through providing good quality colostrum, balanced nutrition, stress minimization; and boosting specific resistance through preventive vaccinations of either the dams or newborn calves against specific pathogens [22,25,26]. Calves in the present study were housed in raised individual wooden hutches up to 60 d of age and maintained on preweaning diets of milk replacer, calf starter grain, and fresh water ad libitum. While a number of calf management practices were in place to reduce calf exposure to risk factors for disease in the current herd, it is possible that some yet unidentified factor (unrelated to pooled MC) may have contributed to the additional risk of diarrhea (e.g. compromised sanitation) leading to increased antimicrobial use on this specific farm.

The overall finding that calves fed raw pooled MC (vs. lacteal-derived CR) were more likely to be treated with antibiotics or experience preweaning diarrhea differed from a previous report [22]. Swan et al. reported no difference in post-feeding morbidity risk observed between calves fed a plasma-derived CR compared with calves fed individual maternal colostrum (i.e. non-pooled colostrum) [22]. In contrast, another study found significantly lower morbidity risk for calves fed MC (46.9%) compared with calves fed a lacteal-derived CR (67.3%) [27]. Although not statistically significant, Priestley et al. reported that a greater proportion of calves fed a lacteal-derived CR experienced diarrhea (44.9%) compared to calves fed MC (28.6%) [27,28]. Although some studies have found no link between FPT and the risk of treatment for diarrhea in beef calves, a possible reason for the difference in findings could be the higher proportion of FPT in calves fed MC in the current study, and in calves fed a lacteal-derived CR (vs. MC) in the Priestley et al. study [27-29]. The difference in FPT is likely to be due to better quality MC fed to calves in the aforementioned studies compared to the current study [22,27]. In a recent survey, 41% of individual colostrum samples met industry recommended colostrum quality benchmarks (IgG >50 g/L and TPC <100,000 cfu/mL) compared with only 26% of pooled colostrum samples [30,31]. The mean IgG mass in the pooled MC fed to the calves in this study was approximately 21 g/L compared with approximately 69.7 g/L of IgG reported for non-pooled colostrum in the recent survey of colostrum quality [24,30].

While there were no differences in the proportion of preweaning respiratory disease, omphalitis or death in calves fed lacteal-derived CR compared to those fed pooled MC, mean daily weight gain was significantly higher in the former. This reflected perhaps a general benefit accruing from the improved preweaning health outcomes associated with feeding lacteal-derived CR rather than a direct role in enhancing daily weight gain in the study calves [2]. Such a link between reduced morbidity and increased weight gain was also reported in a Florida study, although unlike in the current study, the Florida study reported reduced morbidity in calves fed MC compared to those fed a lacteal-derived CR [27,28].

In the current study, type of colostrum fed (CR versus MC) had no effect on mortality risk between birth and weaning in the study calves. These results are consistent with results from an earlier study in which feeding a plasma-derived CR (vs MC) had no effect on preweaning mortality [22]; however they differed from the Florida study which found a significantly lower proportion of mortality in calves fed MC compared to calves fed a lacteal-derived CR [27]. These contradictions may reflect the fact that in concert with passive transfer of immunity, calf survival in the preweaning period is a function of several herd factors including housing and nutrition.

Although a major strength of this study included its randomized assignment of treatment, the results presented here must be interpreted cautiously. It is possible that the ability to correctly assign a disease diagnosis (i.e. diarrhea, respiratory disease, omphalitis) varied among the study personnel involved in the care of the calves during the follow-up period. This potential bias, if present, was likely to be non-differential because the study personnel were blinded to treatment group assignment (lacteal-derived CR vs. pooled MC). Moreover, a veterinarian (SA) cross-validated each recorded diagnosis and treatment with the herd veterinarian’s drug protocols. The extent to which this study’s results can be extrapolated to a wider population of dairy herds is limited by the use of a single herd to evaluate the current study objectives. While approximately 57% of large dairy operations in the US routinely feed pooled MC [31], the current herd was not representative of this wider population with respect to FPT risk profiles and quality of pooled MC fed. Recent findings [32] suggest that approximately 23% of calves in US operations that fed pooled MC experienced FPT compared with 70% of calves fed the pooled MC in this study. In addition, the mean IgG mass in the pooled MC fed to the calves in this study was 3-fold lower than similar estimates of IgG concentration in nationally representative samples of pooled MC [30]. The lower quality of the pooled MC fed to calves in this study compared to comparable US herds likely biased the morbidity outcomes in a direction suggestive of a better efficacy for the lacteal-derived-CR for improving preweaning health in calves. Hence, it is possible that the benefits of feeding lacteal-derived CR (vs. pooled MC) on preweaning health may not be similar in dairy herds managed under a different husbandry system.

The findings reported here indicate that producers should adopt colostrum management strategies that improve colostrum quality. To achieve this, producers can begin by pooling only colostrum with acceptable immunoglobulin concentration (>50 g/liter IgG). This can be achieved by testing individual cow colostrum samples prior to pooling and by properly identifying dams so that first milking colostrum is pooled separate from colostrum harvested from subsequent milkings. Alternatively, pooling colostrum can be replaced by feeding a colostrum replacement product with a known immunoglobulin concentration. Other recommendations identified from previous research should also be followed such as observing proper sanitation and hygiene during harvesting, processing, and storage of colostrum, feeding colostrum replacement products or heat-treated colostrum [2,3,33].

## Conclusions

In this study, calves fed CR had a significantly higher rate of daily weight gain and were less likely to be affected with diarrhea compared to calves fed MC. Type of colostrum fed was not predictive of the preweaning risks for pneumonia and mortality in this particular herd. Although a major strength of this study included its randomized design, the extent to which the current findings can be extrapolated to a wider population of dairy herds may be limited by the use of a single herd.

## Methods

This study was approved by the Institutional Animal Use and Care Committee at the University of California, Davis and the University of Missouri –Columbia (Protocol # 6439).

### Herd selection criteria

This study was conducted in a 3,600 milking Holstein dairy in California that routinely fed 3.8 L of raw pooled MC to newborn calves. The study herd was selected based on its large size, maintenance of electronic records through participation in the California Dairy Herd Information Association routine milk testing and willingness of the owner to comply with the study requirements. Monthly bulk tank milk cultures conducted over the 12 months prior to the study were negative for Mycoplasma species by culture on Hayflick medium and enrichment broth.

### Feeding protocols and experimental design

A total of 568 newborn calves were enrolled in the study between September and December, 2010 while the follow up period ended in March 2011. Pregnant dams were moved to a group calving pen approximately 3 d prior to the expected calving date and monitored until calving. Heifer calves born between 6 AM and midnight were separated from their dams immediately after birth. Cows calving otherwise were monitored by the dairy’s night shift staff and newborn calves were removed immediately after birth to the holding pen until fed the assigned colostrum type by study personnel the following morning. Once in the holding pen, each calf had its navel dipped in an iodine-based antibacterial solution, its ear tagged and its dam ID recorded on farm paper records.

Calves were randomized to one of two treatment groups and fed either 3.8 L of raw pooled MC (control group, n = 273) or two doses of a lacteal-derived CR (Land O Lakes Colostrum Replacement, Lake O Lakes Animal Milk Products) (treatment group, n = 295). The CR was reconstituted based on the manufacturer’s instructions as follows. Each package (100 g IgG/dose) of the lacteal-derived CR powder was dissolved in 1 L of warm water (approx. 49°C) yielding a mixture of 1.4 L containing 71.4 g/L of IgG. Maternal colostrum was harvested after morning milking sessions within 1 to 12 h of calving, pooled and refrigerated at 4°C until feeding within 6–12 h of collection. Both the lacteal-derived CR and pooled MC were administered as a single oral dose (CR ~ 2.8 L and MC ~3.8 L) within 6 h of birth. All calves were fed using a bottle nipple. However, when a calf failed to ingest some or all of the colostrum quantity, an esophageal tube feeder was used to deliver the complete dose in lieu of the bottle.

Following feeding, each calf was housed in an individual raised wooden hutch until weaning at approximately 60 d of age. During the preweaning period, calves were fed daily diets consisting of 2 L of pasteurized hospital pen milk, clean water and a starter grain mix.

### Enrollment and preweaning health monitoring

Before feeding the assigned colostrum type, calves were bled for serum IgG and TP concentration estimation. Calves were also weighed (kg) at birth and weaning which occurred at approximately 60 days of age. Other data recorded included dam identification, colostrum type fed (lacteal-derived CR or pooled MC), birth date, time from birth to separation from dam (h), time from birth to colostrum feeding (h), quantity of colostrum fed (L) and whether or not an esophageal tube feeder was used to administer the colostrum.

Calf morbidity from birth to weaning were monitored and recorded daily by trained study personnel including study authors (SA and JC) while blinded to the type of colostrum fed (lacteal-derived CR or pooled MC) at enrollment. For uniformity in morbidity recordings the following working definitions were applied based on a modification of the University of Wisconsin Calf Health Scoring Chart (http://www.vetmed.wisc.edu/dms/fapm/fapmtools/8calf/calf_health_scoring_chart.pdf): (I) diarrhea was indicated in calves that voided abnormal feces with watery consistency and foul smell (score 2 or 3) with or without dehydration or elevated body temperature (≥ 40°C), (II) respiratory disease was reported for calves that exhibited increased respiratory rate, nasal discharges, or cough (score 1, 2 or 3 except no attempt was made to induce a cough) with or without an elevated body temperature (≥ 40°C), (III) omphalitis (navel infections) was associated with presence of overt umbilical inflammatory signs, including heat, swelling, purulent discharges, or evidence of pain on palpation of the umbilical area. Furthermore, a calf’s joints were physically examined for heat if a joint or umbilicus appeared enlarged. Treatments including antibiotics administered by the dairy’s staff to the study calves were recorded on a daily basis. No specific treatment protocols were developed for this study. Instead, dairy staff relied on protocols implemented by the herd veterinarian. All morbidity events and treatments recorded were validated by a veterinarian (SA).

### Statistical analysis

An estimate for daily weight gain (DWG) for each calf was calculated using the following formula: DWG = (WWT-BWT)/ T; where DWG = Daily weight gain (kg), WWT = weaning weight (kg), BWT = birth weight (kg), T = age of the calf at weaning (d). Mean ± standard error of the mean (SEM) for birth weights, weaning weights, and daily weight gains were calculated. The proportion of calves with an event of diarrhea, respiratory disease, omphalitis, and treated with antibiotics during the preweaning period were calculated for each group (lacteal-derived CR vs. pooled MC). Similarly, the proportion of healthy calves defined as the proportion of calves that did not experience a morbidity event and were not treated with antibiotics from birth to weaning was compared between the study groups. The distribution of the preceding variables between groups (lacteal-derived CR vs. pooled MC) was tested using *t*-tests for continuous variables (birth weight, weaning weights and daily weight gain) and Pearson *χ*^2^-test for categorical variables (diarrhea, respiratory disease, omphalitis, and preweaning treatments with antibiotics, healthy calves, and death).

The association between type of colostrum fed (lacteal-derived CR vs. pooled MC) and diarrhea, respiratory disease, omphalitis, treatment with antibiotics, or producing a healthy calf from birth to weaning were investigated by fitting 5 separate logistic regression models to the data for each outcome. Birth weight and the method by which colostrum was fed (tube vs. bottle feeding) were presented to each model as additional independent variables in a manual forward stepwise selection procedure. Variables that significantly improved model fit based on the Akaike Information Criterion (AIC) values and likelihood ratio test *P* < 0.05 were retained or else excluded from the final models. Population attribution fractions for lacteal-derived CR as a predictor of diarrhea, and treatment with antibiotics in the study calves were estimated separately. Population attributable fractions were estimated using the approach of Greenland and Drescher [34].

Next, to quantify the effect of feeding a lacteal-derived CR (vs. pooled MC) on preweaning time (d) to death, a Cox proportional hazards regression model was fitted to the preweaning mortality data to compare the hazard rate (HR) for mortality in calves fed CR to the HR in calves fed MC using the HRR [35]. Graphical evaluation of the Cox proportional hazard assumption for the treatment group (pooled MC vs. lacteal-derived CR) variable revealed no evidence of violation of the proportional hazards assumption [36].

Finally, a simple least-squares regression model was fitted to the data to evaluate the effect of treatment (lacteal-derived CR (vs. pooled MC) on daily weight gain. Data was analyzed using standard software (Stata^®^ Corp., College Station, TX, USA). A 5% level of significance was indicative of statistical significance.

## Competing interest

Dr. Haines is affiliated with The Saskatoon Colostrum Co. Ltd., the manufacturer of the product kindly donated for the purpose of this study. Her participation did not influence or bias the performance or presentation of the research described in this manuscript in any way.

## Authors’ contributions

SSA, conducted the study, supervised the experiments, performed statistical analysis, and wrote the manuscript. PP conceived the study design, obtained funding for the study, and was actively involved in interpreting the data and writing the manuscript. JDC was actively involved in assisting with supervision of the experiment and data collection. DMH assisted with data interpretation. All authors reviewed and approved the final manuscript.

## References

[B1] Davis CL Drackley JK The development, nutrition, and management of the young calf 1998 Ames, Iowa: Iowa State University Press

[B2] Godden SM Colostrum management for dairy calves Vet Clin North Am Food Anim Pract 2008 24 19 39 10.1016/j.cvfa.2007.10.005 18299030 PMC7127126

[B3] Godden SM Smolenski DJ Donahue M Oakes JM Bey R Wells S Sreevatsan S Stabel J Fetrow J Heat-treated colostrum and reduced morbidity in preweaned dairy calves: results of a randomized trial and examination of mechanisms of effectiveness J Dairy Sci 2012 95 4029 4040 10.3168/jds.2011-5275 22720957

[B4] McGuirk SM Collins M Managing the production, storage, and delivery of colostrum Vet Clin North Am Food Anim Pract 2004 20 593 603 10.1016/j.cvfa.2004.06.005 15471626

[B5] DeNise SK Robison JD Stott GH Armstrong DV Effects of passive immunity on subsequent production in dairy heifers J Dairy Sci 1989 72 552 554 10.3168/jds.S0022-0302(89)79140-2 2703576

[B6] Donovan GA Dohoo IR Montgomery DM Bennett FL Associations between passive immunity and morbidity and mortality in dairy heifers in Florida, USA Prev Vet Med 1998 34 31 46 10.1016/S0167-5877(97)00060-3 9541949 PMC7134088

[B7] Robison JD Stott GH DeNise SK Effects of passive immunity on growth and survival in the dairy heifer J Dairy Sci 1988 71 1283 1287 10.3168/jds.S0022-0302(88)79684-8 3135297

[B8] Virtala AM Grohn YT Mechor GD Erb HN The effect of maternally derived immunoglobulin G on the risk of respiratory disease in heifers during the first 3 months of life Prev Vet Med 1999 39 25 37 10.1016/S0167-5877(98)00140-8 10081786

[B9] Nagy DW Tyler JW Kleiboeker SB Timing of seroconversion and acquisition of positive polymerase chain reaction assay results in calves experimentally infected with bovine leukemia virus Am J Vet Res 2007 68 72 75 10.2460/ajvr.68.1.72 17199421

[B10] Nielsen SS Bjerre H Toft N Colostrum and milk as risk factors for infection with mycobacterium avium subspecies paratuberculosis in dairy cattle J Dairy Sci 2008 91 4610 4615 10.3168/jds.2008-1272 19038936

[B11] Pithua P Godden SM Wells SJ Oakes MJ Efficacy of feeding plasma-derived commercial colostrum replacer for the prevention of transmission of Mycobacterium avium subsp paratuberculosis in Holstein calves J Am Vet Med Assoc 2009 234 1167 1176 10.2460/javma.234.9.1167 19405889

[B12] Steele ML McNab WB Poppe C Griffiths MW Chen S Degrandis SA Fruhner LC Larkin CA Lynch JA Odumeru JA Survey of ontario bulk tank Raw milk for food-borne pathogens J Food Prot 1997 60 1341 1346 10.4315/0362-028X-60.11.1341 31207792

[B13] Streeter RN Hoffsis GF Bech-Nielsen S Shulaw WP Rings DM Isolation of Mycobacterium paratuberculosis from colostrum and milk of subclinically infected cows Am J Vet Res 1995 56 1322 1324 8928949

[B14] Sweeney RW Whitlock RH Rosenberger AE Mycobacterium paratuberculosis cultured from milk and supramammary lymph nodes of infected asymptomatic cows J Clin Microbiol 1992 30 166 171 1734049 10.1128/jcm.30.1.166-171.1992 PMC265014

[B15] Walz PH Mullaney TP Render JA Walker RD Mosser T Baker JC Otitis media in preweaned Holstein dairy calves in Michigan due to Mycoplasma bovis J Vet Diagn Invest 1997 9 250 254 10.1177/104063879700900305 9249163

[B16] Johnson JL Godden SM Molitor T Ames T Hagman D Effects of feeding heat-treated colostrum on passive transfer of immune and nutritional parameters in neonatal dairy calves J Dairy Sci 2007 90 5189 5198 10.3168/jds.2007-0219 17954759

[B17] Quigley JD Strohbehn RE Kost CJ O’Brien MM Formulation of colostrum supplements, colostrum replacers and acquisition of passive immunity in neonatal calves J Dairy Sci 2001 84 2059 2065 10.3168/jds.S0022-0302(01)74650-4 11573786 PMC7157919

[B18] Chelack BJ Morley PS Haines DM Evaluation of methods for dehydration of bovine colostrum for total replacement of normal colostrum in calves Can Vet J 1993 34 407 412 17424250 PMC1686489

[B19] Fidler AP Alley ML Smith GW Short communication: serum immunoglobulin G and total protein concentrations in dairy calves fed a colostrum-replacement product J Dairy Sci 2011 94 3609 3612 10.3168/jds.2011-4358 21700049

[B20] Foster DM Smith GW Sanner TR Busso GV Serum IgG and total protein concentrations in dairy calves fed two colostrum replacement products J Am Vet Med Assoc 2006 229 1282 1285 10.2460/javma.229.8.1282 17042734

[B21] Godden SM Haines DM Hagman D Improving passive transfer of immunoglobulins in calves. I: dose effect of feeding a commercial colostrum replacer J Dairy Sci 2009 92 1750 1757 10.3168/jds.2008-1846 19307657

[B22] Swan H Godden S Bey R Wells S Fetrow J Chester-Jones H Passive Transfer of Immunoglobulin G and Preweaning Health in Holstein Calves Fed a Commercial Colostrum Replacer J Dairy Sci 2007 90 3857 3866 10.3168/jds.2007-0152 17638996

[B23] Poulsen KP Foley AL Collins MT McGuirk SM Comparison of passive transfer of immunity in neonatal dairy calves fed colostrum or bovine serum-based colostrum replacement and colostrum supplement products J Am Vet Med Assoc 2010 237 949 954 10.2460/javma.237.8.949 20946083 PMC3042274

[B24] Pithua P Aly SS Haines DM Champagne JD Middleton JR Poock SE Efficacy of feeding a lacteal-derived colostrum replacer or pooled maternal colostrum with a low IgG concentration for prevention of failure of passive transfer in dairy calves J Am Vet Med Assoc 2013 243 277 282 10.2460/javma.243.2.277 23822086

[B25] Davis R Giguere S Evaluation of five commercially available assays and measurement of serum total protein concentration via refractometry for the diagnosis of failure of passive transfer of immunity in foals J Am Vet Med Assoc 2005 227 1640 1645 10.2460/javma.2005.227.1640 16313044

[B26] Pithua P Wells SJ Godden SM Raizman EA Clinical trial on type of calving pen and the risk of disease in Holstein calves during the first 90 d of life Prev Vet Med 2009 89 8 15 10.1016/j.prevetmed.2009.01.001 19193464 PMC7172793

[B27] Priestley D Bittar JH Ibarbia L Risco CA Galvao KN Effect of feeding maternal colostrum or plasma-derived or colostrum-derived colostrum replacer on passive transfer of immunity, health, and performance of preweaning heifer calves J Dairy Sci 2013 96 3247 3256 10.3168/jds.2012-6339 23497992

[B28] Waldner CL Rosengren LB Factors associated with serum immunoglobulin levels in beef calves from Alberta and Saskatchewan and association between passive transfer and health outcomes Can Vet J 2009 50 275 281 19436479 PMC2643452

[B29] Filteau V Bouchard E Fecteau G Dutil L DuTremblay D Health status and risk factors associated with failure of passive transfer of immunity in newborn beef calves in Quebec Can Vet J 2003 44 907 913 14664353 PMC385448

[B30] Morrill KM Conrad E Lago A Campbell J Quigley J Tyler H Nationwide evaluation of quality and composition of colostrum on dairy farms in the United States J Dairy Sci 2012 95 3997 4005 10.3168/jds.2011-5174 22720954

[B31] NAHMS CEAH UAV Heifer Calf Health and Management Practices on U.S. Dairy Operations, 2007 2010 Fort Collins, CA:

[B32] Beam AL Lombard JE Kopral CA Garber LP Winter AL Hicks JA Schlater JL Prevalence of failure of passive transfer of immunity in newborn heifer calves and associated management practices on US dairy operations J Dairy Sci 2009 92 3973 3980 10.3168/jds.2009-2225 19620681

[B33] Stewart S Godden S Bey R Rapnicki P Fetrow J Farnsworth R Scanlon M Arnold Y Clow L Mueller K Preventing bacterial contamination and proliferation during the harvest, storage, and feeding of fresh bovine colostrum J Dairy Sci 2005 88 2571 2578 10.3168/jds.S0022-0302(05)72933-7 15956318

[B34] Greenland S Drescher K Maximum likelihood estimation of the attributable fraction from logistic models Biometrics 1993 49 865 872 10.2307/2532206 8241375

[B35] Cox DR Regression models and life-tables J R Stat Soc Series B 1972 34 187 220

[B36] Cleves AC Gould WW Gutierrez RG An introduction to survival analysis using stata 2004 College Station, Texas: Stata Press

